# Supplementary material for: A Systematic Review and Meta-Analysis of Glyphosate-Based Herbicide Exposure and Risk of Non-Hodgkin Lymphoma
Source: Int J Environ Res Public Health. 2026 Jul 30;23(8):1000. doi: 10.3390/ijerph23081000 (PMC13512628; doi:10.3390/ijerph23081000)
Supplement: Supplementary file 1 [file ijerph-23-01000-s001.zip › ijerph-4447356-supplementary-figures.pdf]

Figure S1: Ever-exposure data removing Leon and Poh: Fixed Effects Forest Plot

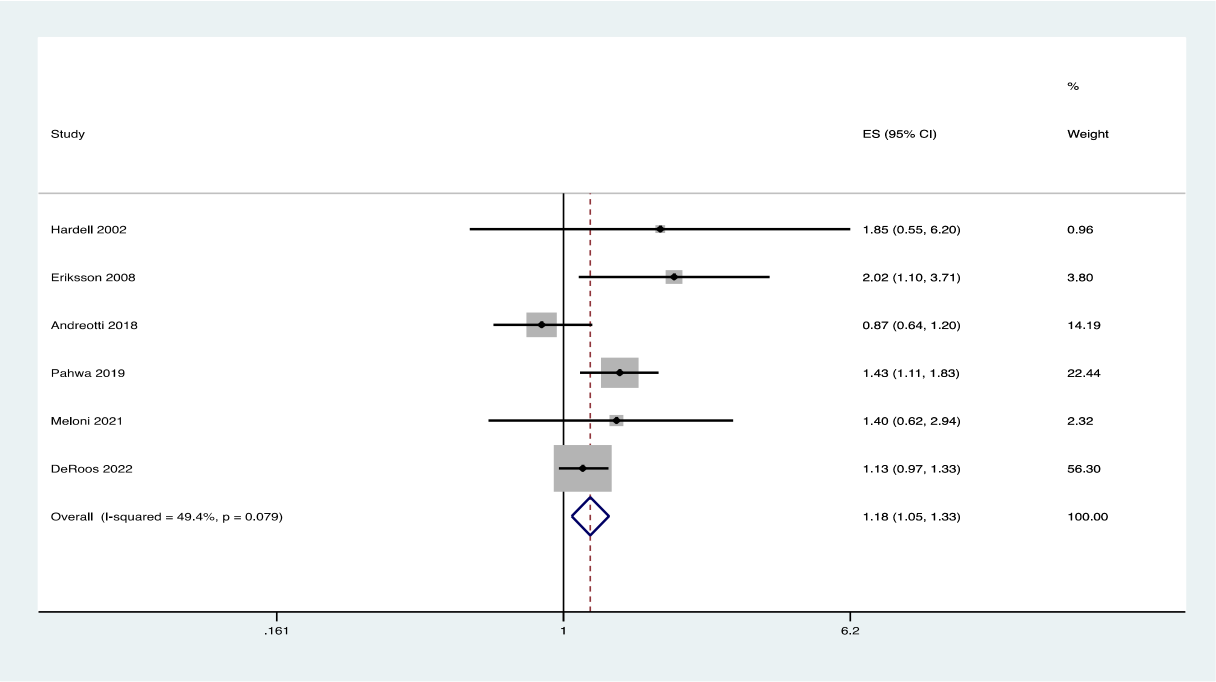

Figure S2: Ever-exposure data removing Leon and Poh: Random Effects Forest Plot

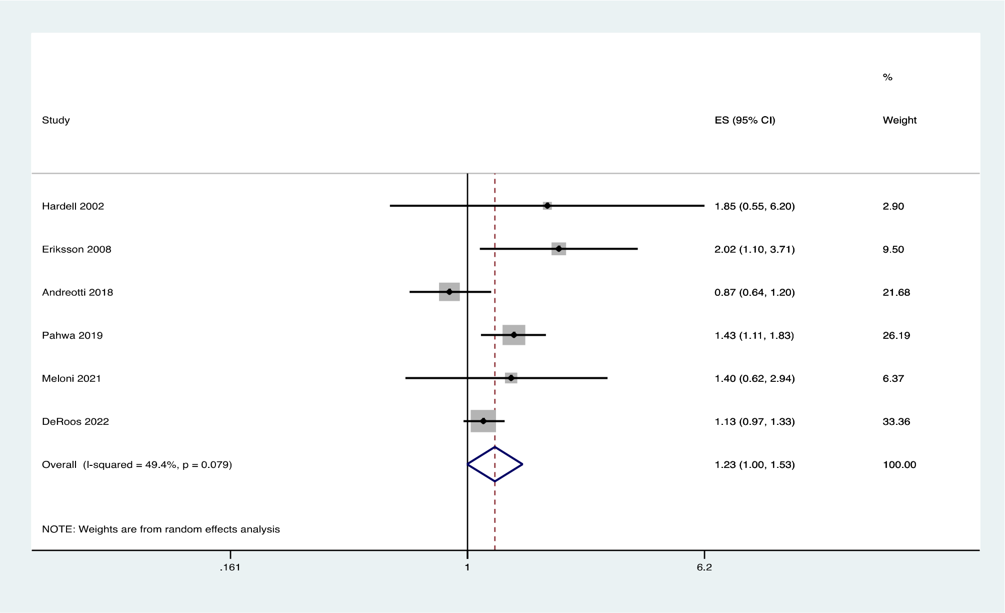

Figure S3: Random Effects Meta-Analysis Results Table for Highest Exposure Category

```
. metan LogOR LogLCI LogUCI, eform randomi lcols (StudyID)

           Study     |     ES    [95% Conf. Interval]     % Weight
---------------------+---------------------------------------------------
Hardell 2002         |  1.850       0.550     6.200          2.43
Eriksson 2008        |  2.360       1.040     5.370          4.80
Andreotti 2018       |  1.120       0.830     1.510         17.26
Leon 2019            |  0.950       0.770     1.180         21.44
Pahwa 2019           |  2.420       1.480     3.960         10.29
Meloni 2021          |  1.100       0.350     3.510          2.65
Poh 2022             |  1.000       0.920     1.080         27.35
DeRoos 2022          |  1.075       0.733     1.576         13.78
---------------------+---------------------------------------------------
D+L pooled ES        |  1.183       0.971     1.441        100.00
---------------------+---------------------------------------------------
 Heterogeneity calculated by formula
  Q = SIGMA_i{ (1/variance_i)*(effect_i - effect_pooled)^2 }
where variance_i = ((upper limit - lower limit)/(2*z))^2

  Heterogeneity chi-squared =  17.83 (d.f. = 7) p = 0.013
  I-squared (variation in ES attributable to heterogeneity) =  60.7%
  Estimate of between-study variance Tau-squared =  0.0353

  Test of ES=1 : z=   1.67 p = 0.095
```

Figure S4: Forest Plot Random Effects Meta-Analysis for highest exposure category

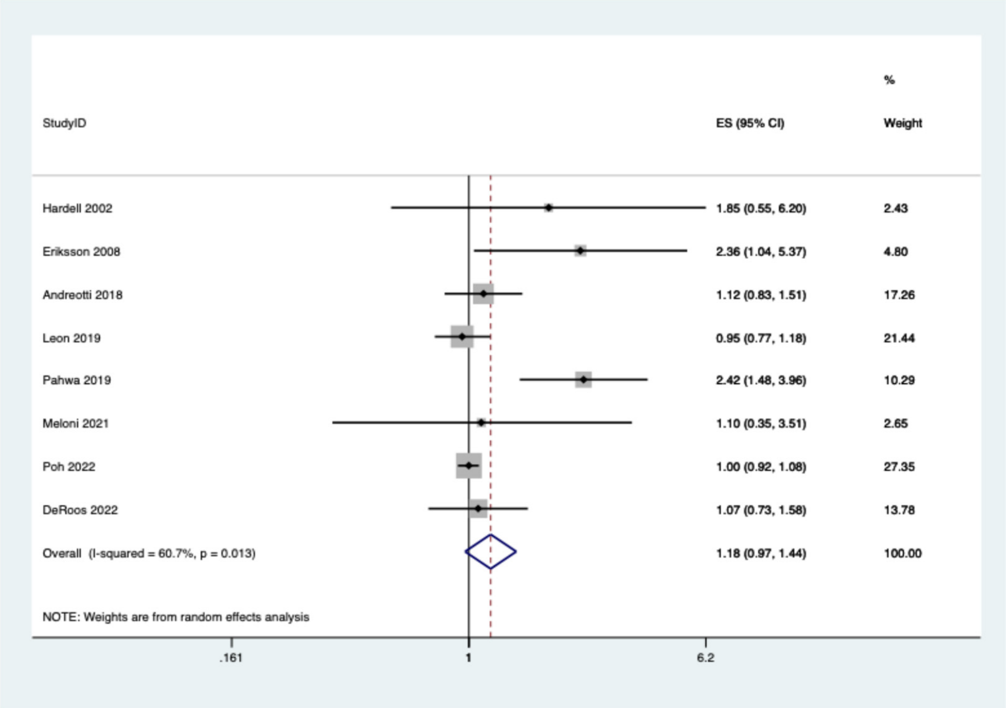

Figure S5: Random Effects Meta-Analysis Results Table for Highest Exposure Category removing Leon and Poh

```
. metan LogOR LogLCI LogUCI, eform lcols (Study) randomi

           Study     |     ES    [95% Conf. Interval]     % Weight
---------------------+---------------------------------------------------
Hardell 2002         |  1.850      0.550     6.200          6.48
Eriksson 2008        |  2.360      1.040     5.370         11.70
Andreotti 2018       |  1.120      0.830     1.510         28.77
Pahwa 2019           |  2.420      1.480     3.960         20.83
Meloni 2021          |  1.100      0.350     3.510          7.03
DeRoos 2022          |  1.075      0.733     1.576         25.19
---------------------+---------------------------------------------------
D+L pooled ES        |  1.465      1.043     2.057        100.00
---------------------+---------------------------------------------------
 Heterogeneity calculated by formula
  Q = SIGMA_i{ (1/variance_i)*(effect_i - effect_pooled)^2 }
where variance_i = ((upper limit - lower limit)/(2*z))^2

  Heterogeneity chi-squared =  10.41 (d.f. = 5) p = 0.064
  I-squared (variation in ES attributable to heterogeneity) =  52.0%
  Estimate of between-study variance Tau-squared =  0.0809

  Test of ES=1 : z=   2.21 p = 0.027
```

Figure S6: Forest Plot Random Effects Meta-Analysis for highest exposure category removing Leon and Poh

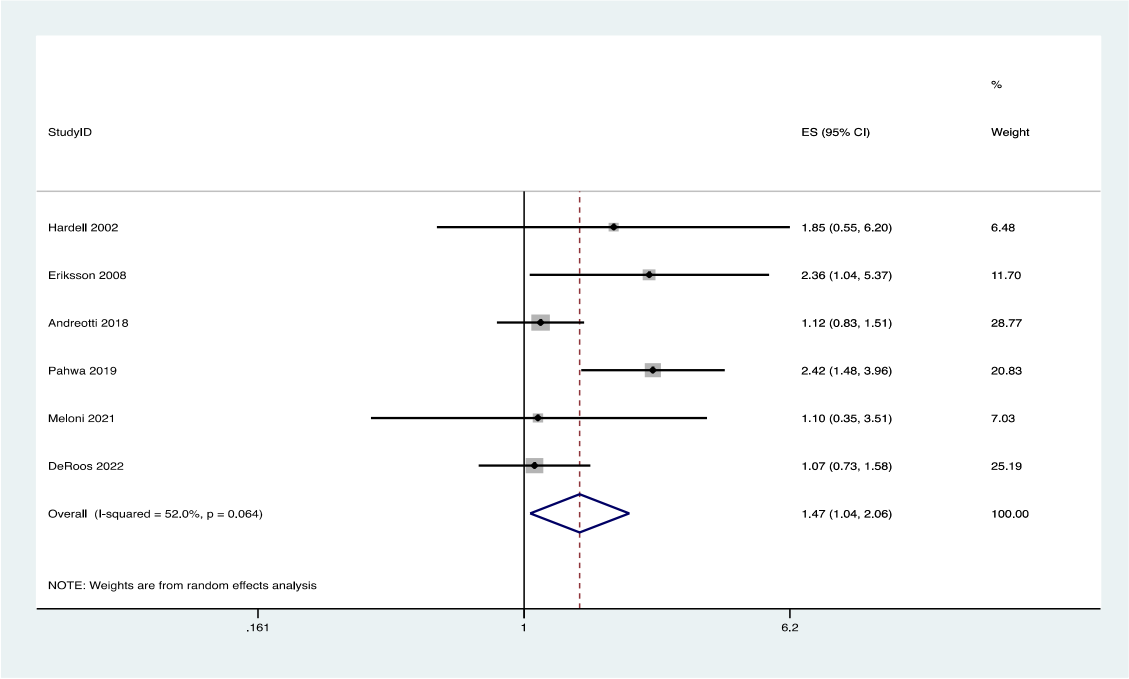

Figure S7: Fixed Effects Meta-Analysis Results Table for Highest Exposure Category with Cumulative Exposure from Meloni and Pahwa removing Leon and Poh

```
. metan LogOR LogLCI LogUCI, eform lcols (Study)

          Study       |     ES    [95% Conf. Interval]     % Weight
---------------------+---------------------------------------------------
Hardell 2002         |  1.850       0.550     6.200          4.13
Eriksson 2008        |  2.360       1.040     5.370          9.00
Orsi 2009            |  1.010       0.500     2.200         11.04
Meloni 2021          |  1.500       0.490     4.810          4.65
DeRoos 2022          |  1.075       0.733     1.576         41.39
Pahwa 2019           |  1.550       0.990     2.440         29.79
---------------------+---------------------------------------------------
I-V pooled ES        |  1.327       1.037     1.697        100.00
---------------------+---------------------------------------------------
 Heterogeneity calculated by formula
  Q = SIGMA_i{ (1/variance_i)*(effect_i - effect_pooled)^2 }
where variance_i = ((upper limit - lower limit)/(2*z))^2

  Heterogeneity chi-squared =   4.37 (d.f. = 5) p = 0.498
  I-squared (variation in ES attributable to heterogeneity) =   0.0%

  Test of ES=1 : z=   2.25 p = 0.024
```

Figure S8: Forest Plot Fixed Effects Meta-Analysis for highest exposure category with Cumulative Exposure from Meloni and Pahwa removing Leon and Poh

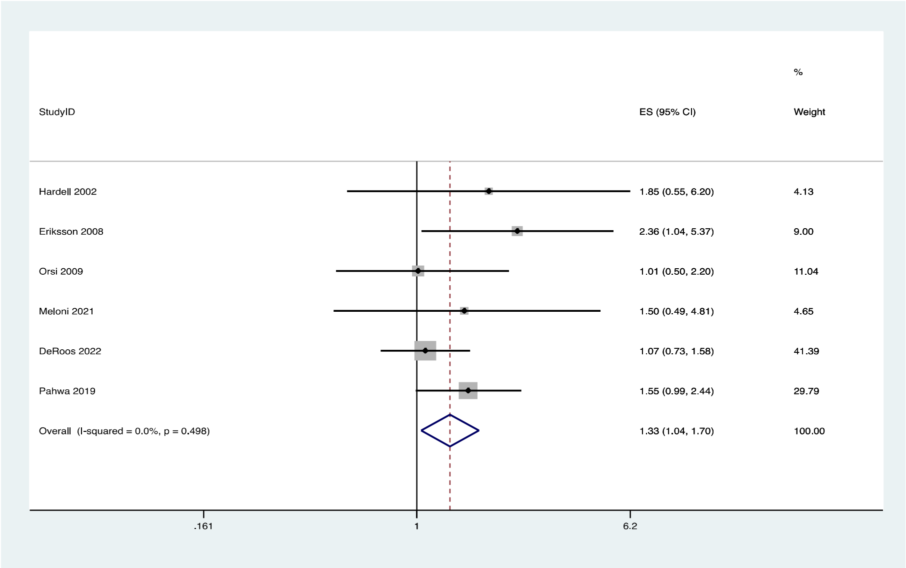

Figure S9: Standard funnel plot for ever-exposure data for all non-duplicate included studies.

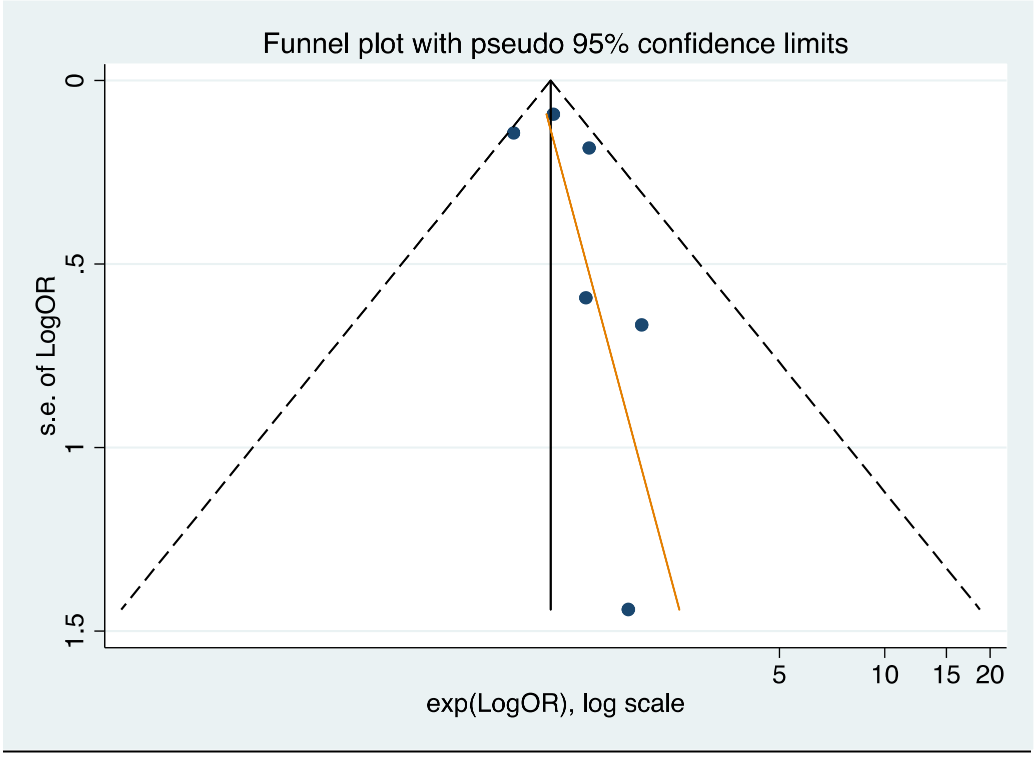

Figure S10: Contour-enhanced plot for ever-exposure data for all non-duplicate included studies.

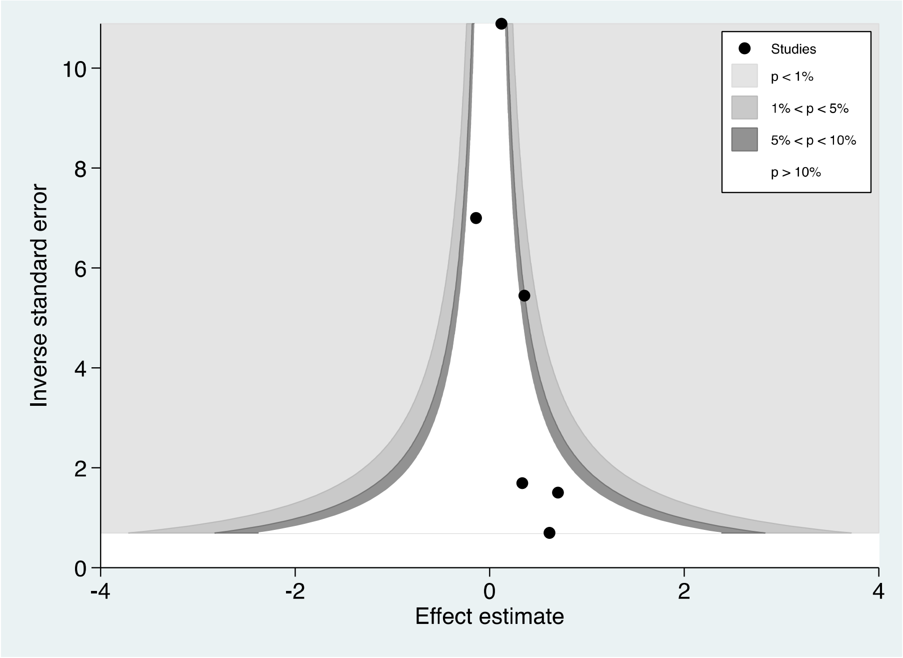

Figure S11: Standard funnel plot for highest level of exposure data for all non-duplicate included studies.

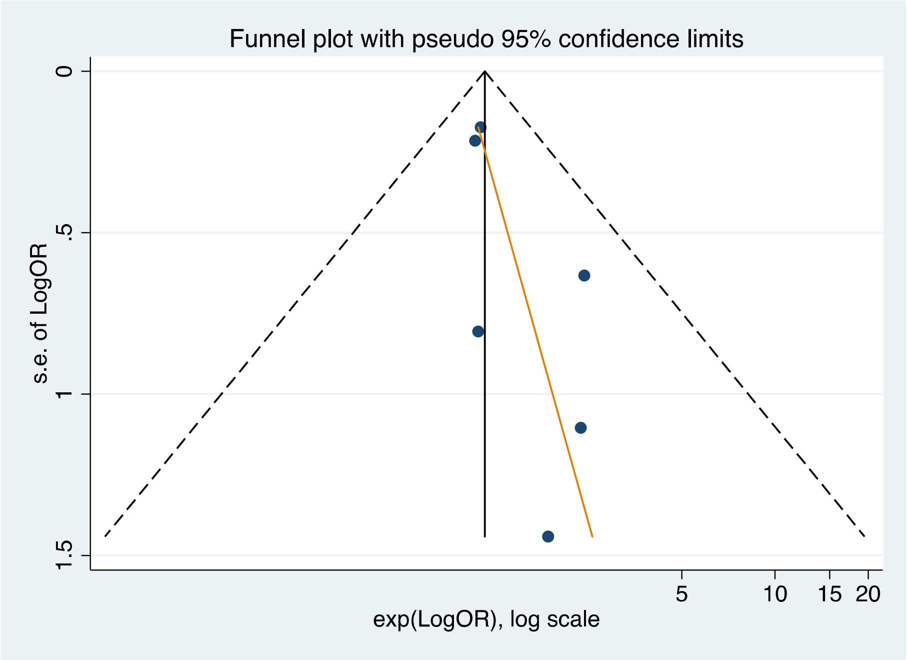

Figure S12: Contour-enhanced funnel plot for highest level of exposure data for all non-duplicate included studies.

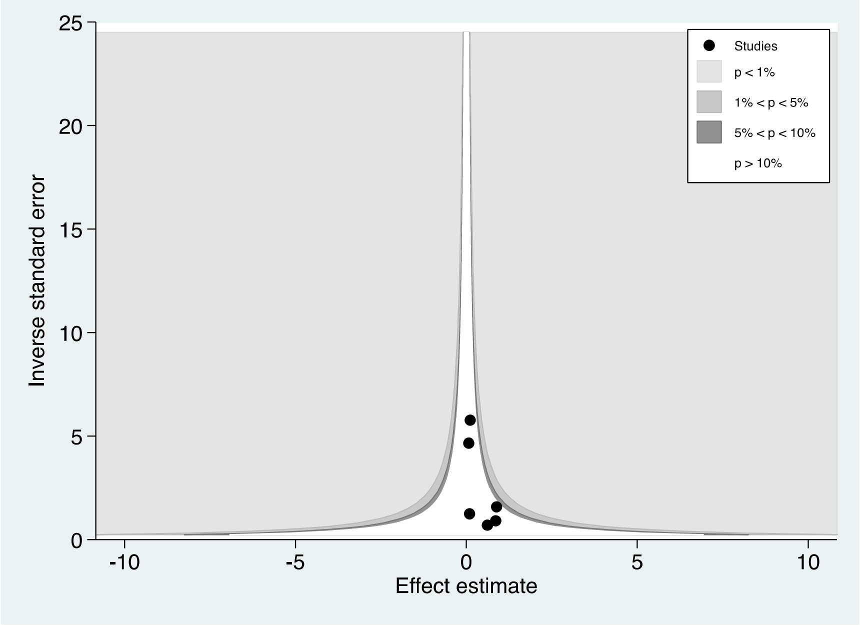

Figure S13: Meta-regression exploring variation in ever-exposed meta-analytic estimate by risk of bias

```
Meta-regression                                      Number of obs  =        6
REML estimate of between-study variance              tau2           =   .06813
% residual variation due to heterogeneity            I-squared_res  =   51.33%
Proportion of between-study variance explained       Adj R-squared  =  -52.95%
With Knapp-Hartung modification
```

OR	Coef.	Std. Err.	t	P>|t|	[95% Conf.	Interval]
ROB	.0026606	.0102318	0.26	0.808	-.0257475	.0310687
_cons	1.022292	.7019804	1.46	0.219	-.9267177	2.971302

Figure S14: Meta-regression exploring variation in highest exposure meta-analytic estimate by risk of bias

```
Meta-regression                                      Number of obs  =        6
REML estimate of between-study variance              tau2           =    .1077
% residual variation due to heterogeneity            I-squared_res  =   18.38%
Proportion of between-study variance explained       Adj R-squared  =       .%
With Knapp-Hartung modification
```

OR	Coef.	Std. Err.	t	P>|t|	[95% Conf.	Interval]
ROB_01	.0137842	.0145249	0.95	0.396	-.0265434	.0541118
_cons	.4098573	.9792562	0.42	0.697	-2.308994	3.128708

References

6. Eriksson, M.; Hardell, L.; Carlberg, M.; Akerman, M. Pesticide exposure as risk factor for non-Hodgkin lymphoma including histopathological subgroup analysis. *Int. J. Cancer* **2008**, *123*, 1657–1663.
7. Hardell, L.; Eriksson, M.; Nordstrom, M. Exposure to pesticides as risk factor for non-Hodgkin lymphoma and hairy cell leukemia: Pooled analysis of 2 Swedish case-control studies. *Leuk. Lymphoma* **2002**, *43*, 1043–1049.
8. McDuffie, H.H.; Pahwa, P.; McLaughlin, J.R.; Spinelli, J.J.; Fincham, S.; Dosman, J.A.; Robson, D.; Skinnider, L.F.; Choi, N.W. Non-Hodgkin lymphoma and specific pesticide exposures in men: Cross-Canada study of pesticides and health. *Cancer Epidemiol. Biomark. Prev.* **2001**, *10*, 1155–1163.

9. Orsi, L.; Delabre, L.; Monnereau, A.; Delval, P.; Berthou, C.; Fenaux, P.; Marit, G.; Soubeyran, P.; Huguet, F.; Milpied, N.; et al. Occupational exposure to pesticides and lymphoid neoplasms among men: Results of a French case-control study. *Occup. Environ. Med.* **2009**, *66*, 291–298.
18. Meloni, F.; Satta, G.; Padoan, M.; Montagna, A.; Pilia, I.; Argiolas, A.; Piro, S.; Magnani, C.; Gambelunghe, A.; Muzi, G.; et al. Occupational exposure to glyphosate and risk of lymphoma: Results of an Italian multicenter case-control study. *Environ. Health* **2021**, *20*, 49. https://doi.org/10.1186/s12940-021-00729-8.
19. Poh, C.; McPherson, J.D.; Tuscano, J.; Li, Q.; Parikh-Patel, A.; Vogel, C.F.A.; Cockburn, M.; Keegan, T. Environmental pesticide exposure and non-Hodgkin lymphoma survival: A population-based study. *BMC Med.* **2022**, *20*, 165.
20. De Roos, A.J.; Fritschi, L.; Ward, M.H.; Monnereau, A.; Hofmann, J.; Bernstein, L.; Bhatti, P.; Benavente Moreno, Y.; Benke, G.; Casabonne, D.; et al. Herbicide use in farming and other jobs in relation to non-Hodgkin lymphoma risk. *Occup. Environ. Med.* **2022**, *79*, 795–806.
39. Andreotti, G.; Koutros, S.; Hofmann, J.N.; Sandler, D.P.; Lubin, J.H.; Lynch, C.F.; Lerro, C.C.; De Roos, A.J.; Parks, C.G.; Alavanja, M.C.; et al. Glyphosate use and cancer incidence in the Agricultural Health Study. *J. Natl. Cancer Inst.* **2018**, *110*, 509–516.
44. Leon, M.E.; Beane Freeman, L.E.; Douwes, J.; Hoppin, J.A.; Kromhout, H.; Lebailly, P.; Nordby, K.C.; Schenker, M.; Schüz, J.; Waring, S.C.; et al. AGRICOH: A consortium of agricultural cohorts. *Int. J. Environ. Res. Public Health* **2011**, *8*, 1341–1357.
46. Pahwa, M.; Beane Freeman, L.E.; Spinelli, J.J.; Blair, A.; McLaughlin, J.R.; Zahm, S.H.; Cantor, K.P.; Weisenburger, D.D.; Pahwa, P.; Dosman, J.A.; et al. Glyphosate use and associations with non-Hodgkin lymphoma major histological subtypes: Findings from the North American Pooled Project. *Scand. J. Work. Environ. Health* **2019**, *45*, 600–609. https://doi.org/10.5271/sjweh.3830.
